# “Doing the Impossible with the Inadequate”: COVID-19 Response in U.S. Assisted Living Settings

**DOI:** 10.1093/geroni/igaa057.3412

**Published:** 2020-12-16

**Authors:** Sarah Dys, Jaclyn Winfree, Paula Carder, Kaylin Dugle, Sheryl Zimmerman, Kali Thomas

**Affiliations:** 1 Institute on Aging, Portland, Oregon, United States; 2 Portland State University, Portland, Oregon, United States; 3 University of North Carolina at Chapel Hill, Chapel Hill, North Carolina, United States; 4 Brown University, Providence, Rhode Island, United States

## Abstract

The COVID-19 pandemic has disproportionately affected long-term care operators, staff, residents and their families; although much attention has been given to nursing homes, largely lost in the discourse are assisted living, residential care, and dementia care (AL) communities. As part of a broader, ongoing study assessing states’ AL regulations regarding medical and mental health care for residents with Alzheimer’s and related dementias (ADRD), stakeholders across the United States were recruited in July and August 2020 for semi-structured interviews to provide their perspectives on AL policies and practices specific to COVID-19 response. Stakeholders (n=32) consisted of state healthcare and trade association representatives, clinical practitioners, operators, researchers, and dementia care experts experienced in AL-related operational, healthcare, and regulatory affairs. Using thematic analysis, we describe several emerging topics regarding the opportunities, challenges, and innovations of responding to COVID-19 within the unique context of AL. States’ public health responses to COVID-19 lacked an understanding of the broader long-term care system, especially AL’s scope and purpose, workforce, capacity to implement infection control practices and policies, and unintended consequences of social isolation for older adults, specifically residents living in dementia care units. Despite these challenges, stakeholders described opportunities to expand telehealth infrastructure, communication and collaboration across states and among operators, and several innovations to mitigate the effects of social isolation. It is imperative for policymakers to understand the nuances of the AL context and design regulations and public health responses grounded in a whole-person perspective and in partnership with operators during, and beyond, pandemic circumstances.

